# Expanded noninvasive prenatal testing for fetal aneuploidy and copy number variations and parental willingness for invasive diagnosis in a cohort of 18,516 cases

**DOI:** 10.1186/s12920-021-00955-6

**Published:** 2021-04-14

**Authors:** Yunsheng Ge, Jia Li, Jianlong Zhuang, Jian Zhang, Yanru Huang, Meihua Tan, Wei Li, Jiayan Chen, Yulin Zhou

**Affiliations:** 1 Prenatal Diagnosis Center, Women and Children’s Hospital, School of Medicine, Xiamen University, 10 Zhenhai Road, Xiamen, 361003 Fujian Province China; 2 BGI-Genomics, BGI-Shenzhen, Shenzhen, Guangdong Province China; 3 Prenatal Diagnosis Center, Quanzhou Women’s and Children’s Hospital, Quanzhou, 362000 Fujian Province China

**Keywords:** Expanded noninvasive prenatal test, Common trisomies, Sex chromosomal aneuploidies, Rare autosomal aneuploidies, Copy number variations

## Abstract

**Background:**

Noninvasive prenatal testing (NIPT) has been wildly used to screen for common aneuplodies. In recent years, the test has been expanded to detect rare autosomal aneuploidies (RATs) and copy number variations (CNVs). This study was performed to investigate the performance of expanded noninvasive prenatal testing (expanded NIPT) in screening for common trisomies, sex chromosomal aneuploidies (SCAs), rare autosomal aneuploidies (RATs), and copy number variations (CNVs) and parental willingness for invasive prenatal diagnosis in a Chinese prenatal diagnosis center.

**Methods:**

A total of 24,702 pregnant women were retrospectively analyzed at the Women and Children’s Hospital from January 2013 to April 2019, among which expanded NIPT had been successfully conducted in 24,702 pregnant women. The high-risk expanded NIPT results were validated by karyotype analysis and chromosomal microarray analysis. All the tested pregnant women were followed up for pregnancy outcomes.

**Results:**

Of the 24,702 cases, successful follow-up was conducted in 98.77% (401/446) of cases with common trisomies and SCAs, 91.95% (80/87) of RAT and CNV cases, and 76.25% (18,429/24,169) of cases with low-risk screening results. The sensitivity of expanded NIPT was 100% (95% confidence interval[CI], 97.38–100%), 96.67%(95%CI, 82.78–99.92%), and 100%(95%CI, 66.37–100.00%), and the specificity was 99.92%(95%CI, 99.87–99.96%), 99.96%(95%CI, 99.91–99.98%), and 99.88% (95%CI, 99.82–99.93%) for the detection of trisomies 21, 18, and 13, respectively. Expanded NIPT detected 45,X, 47,XXX, 47,XXY, XYY syndrome, RATs, and CNVs with positive predictive values of 25.49%, 75%, 94.12%, 76.19%, 6.45%, and 50%, respectively. The women carrying fetuses with Trisomy 21/Trisomy 18/Trisomy 13 underwent invasive prenatal diagnosis and terminated their pregnancies at higher rates than those at high risk for SCAs, RATs, and CNVs.

**Conclusions:**

Our study demonstrates that the expanded NIPT detects fetal trisomies 21, 18, and 13 with high sensitivity and specificity. The accuracy of detecting SCAs, RATs, and CNVs is still relatively poor and needs to be improved. With a high-risk expanded NIPT result, the women at high risk for common trisomies are more likely to undergo invasive prenatal diagnosis procedures and terminate their pregnancies than those with unusual chromosome abnormalities.

**Supplementary Information:**

The online version contains supplementary material available at 10.1186/s12920-021-00955-6.

## Background

Noninvasive prenatal testing (NIPT) was first introduced to screen for fetal Trisomy 21 (T21) in 2011 and went global rapidly [1]. To date, NIPT has been conducted in millions of cases throughout the world, and the clinical performance of expanded NIPT to detect fetal T21, Trisomy 18 (T18), Trisomy 13 (T13), and sex chromosomal aneuploidies (SCAs) are now well recognized [2–6]. In recent years, the test has been expanded to detect rare autosomal aneuploidies (RATs) and copy number variations (CNVs) [7–11]. Many factors affect the decisions to pursue further prenatal diagnosis and to continue or terminate pregnancy, including the type of fetal disease, prenatal genetic counseling, the quality of life of the pregnant woman, cultural factors, multiple cross-disciplinary approaches, etc. [12].

Despite significant progresses in the application of NIPT, there are several problems which are poorly understood. First of all, it remains unclear regarding the detection accuracies of RATs and CNVs of the expanded NIPT, which restricts the wide application of expanded NIPT to screen for RATs and CNVs in clinical settings; Secondly, upon receiving different high-risk expanded NIPT results, the attitude of pregnant women towards invasive prenatal diagnosis and termination of pregnancy remains unknown. Therefore, a systematic review of expanded NIPT results, diagnostic tests, and pregnancy outcomes of all screening results is needed in a large-scale population, to enable comprehensively evaluation of the clinical performance of the test and parental willingness for invasive prenatal diagnosis.

In the present study, we analyzed the clinical data of a cohort of 24,702 participants who underwent expanded NIPT at the Women and Children’s Hospital from January 2013 to April 2019 in Xiamen of Fujian Province, China. We reviewed the expanded NIPT results, the diagnostic invasive test results, and clinical follow-up information to evaluate the performance of expanded NIPT in screening for T21, T18, T13, SCAs, RATs, and CNVs. We also retrospectively analyzed the clinical data of 533 pregnant women with high-risk expanded NIPT results and examined their clinical treatments, the results of prenatal diagnosis, and pregnancy outcomes to study their willingness for invasive prenatal diagnosis and termination of their pregnancies.

## Methods and materials

### Ethics statement and sample collection

This was a retrospective study. A total of 24,702 participants were retrospectively analyzed from the Women and Children’s Hospital from January 2013 to April 2019 in Xiamen of Fujian Province, China. All the participants provided written informed consent. This study followed strict confidentiality regulations on privacy protection and was approved by the Institutional Review Board of the Women and Children’s Hospital (KY-2019-037). Five milliliters of peripheral blood were taken from the pregnant woman and stored in Streck Cell Free DNA BCT ® blood collection tubes (Streck, La Vista, Nebraska, United States), and plasma DNA was extracted within four days after collection.

### Sequencing and bioinformatic analysis

Details of the expanded NIPT methods have been published previously [13, 14]. In brief, plasma was separated by sequential centrifugations of the blood sample at 1600 g at 4 °C for 10 min. Cell-free DNA was extracted from plasma and subjected to library construction. The quantity and quality of the library were examined by real-time polymerase chain reaction and size distribution analysis. Only the qualified library was sequenced, and the data generated were analyzed using the Noninvasive Fetal Trisomy (NIFTY) algorithm to detect T21, T18, T13, SCAs, RATs as previously described [13]. The Fetal Copy-number Analysis through Maternal Plasma Sequencing (FCAPS) method was used for noninvasive genome-wide detection of fetal large deletions/duplications [14].

### Validation of high-risk screening results

Participants who received high-risk results of T21, T18, T13, SCAs, and RATs were recommended to take confirmatory invasive tests using amniocentesis followed by karyotype analysis. For cases with high-risk CNV results, chromosomal microarray analysis (CMA) was performed for validation. Prenatal diagnosis was carried out according to our routine experimental method and completed in our prenatal diagnosis center.

### Clinical follow-up analysis

Routine prenatal care was recommended for participants who had low-risk screening results in expanded NIPT. Ultrasound examination was performed regularly to monitor fetal growth or detected anomalies to monitor fetal progression. If abnormal fetal anomalies were observed, participants were offered genetic counselling. For the other participants who declined termination of pregnancy (TOP), a postnatal follow-up was conducted by telephone interview at 3 months after birth. The following information was collected, including the ultrasound examination report, final pregnancy outcomes, baby’s sex, and newborn physical examination results.

### Evaluation of the performance of expanded NIPT

Pregnancies who had high-risk expanded NIPT results for T21, T18, T13, SCAs, RATs, and CNVs were recommended for diagnostic tests using amniocentesis. True positive and false positive referred to the high-risk expanded NIPT results that were concordant or discordant with diagnostic genetic testing, respectively. The true-negative cases were low risk in expanded NIPT and validated as normal by neonatal physical examination, except for SCAs or diagnostic test analysis. False negative referred to those cases reported to be low-risk for common aneuploidies but presented an aneuploidy karyotype which was validated by invasive genetic testing. Notably, the true-positive and false-positive CNVs were validated by the gold-standard CMA analysis. The total number of cases used to calculate the performance of NIPT in the screening for common aneuploidies is 18,516, comprising 18,028 low-risk cases with live births, 100 low-risk cases with diagnostic results, 339 high-risk cases with diagnostic results, 33 cases who had high risk RAT and diagnostic results, and 16 cases who had high risk CNV and diagnostic results. Participants who did not have confirmatory diagnostic results, low-risk cases with pregnancy losses, cases with TOP, stillbirths, cases with unknown abnormalities, and cases lost to follow-up were excluded in the study. Loss to follow-up referred to cases that were unreachable or rejected to be interviewed. The sensitivity, specificity, positive predictive value (PPV) and negative predictive value (NPV) were calculated for the detection of common aneuploidies in a cohort of 18,516 cases. PPVs were computed accordingly for the detection of SCAs, RATs and CNVs.

### Comparison of prenatal diagnosis willingness and pregnancy outcomes

For the pregnant women who are at high risk for common aneuploidies, SCAs, RATs and CNVs, the number of women who accepted or declined invasive prenatal diagnosis was respectively counted. Moreover, for the cases confirmed to be high risk for common aneuploidies, SCAs, RATs and CNVs, the number of women who chose TOP or continuation of pregnancy were counted. The willingness towards prenatal diagnosis and pregnancy outcomes were compared for pregnant women with high-risk results of common aneuploidies, SCAs, RATs and CNVs separately using Fisher’s exact test in R3.2.0, *p* values ≤ 0.05 were considered statistically significant.

## Results

### General characteristics of expanded NIPT results

The mean maternal age of the 24,702 cases was 32.53 years old, and 30.9% (7628/24,702) of the cases had maternal age greater than 35 years old at the time of expanded NIPT. The gestational weeks ranged from 12 to 33 weeks, with a mean of 16.87 weeks. Of the 24,702 cases, 1.8% (446/24,702) of cases were found to be high risk for T21, T18, T13, and SCAs. Additionally, 0.22% (54/24,702) and 0.13% (33/24,702) of cases were at high risk for RATs and CNVs, respectively. The incidences of common trisomies in pregnant women with older maternal age (> 35 years) were significantly higher than those in pregnant women with younger maternal age (≤ 35 years) [*p* < 0.05, 2.16%(165/7628) vs. 1.64%(281/17,074), Fisher’s exact test]. While, the incidences of RATs and CNVs were not statistically different between older pregnant women (age > 35 years) and young ones (age ≤ 35 years) [*p* = 0.66, 0.24%(18/7628) vs. 0.21%(36/17,074) for RATs; and *p* = 1, 0.13%(10/7628) vs. 0.13%(23/17,074) for CNVs, Fisher’s exact test].

### Expanded NIPT results for T21, T18, T13, and SCAs

**Fig. 1:**
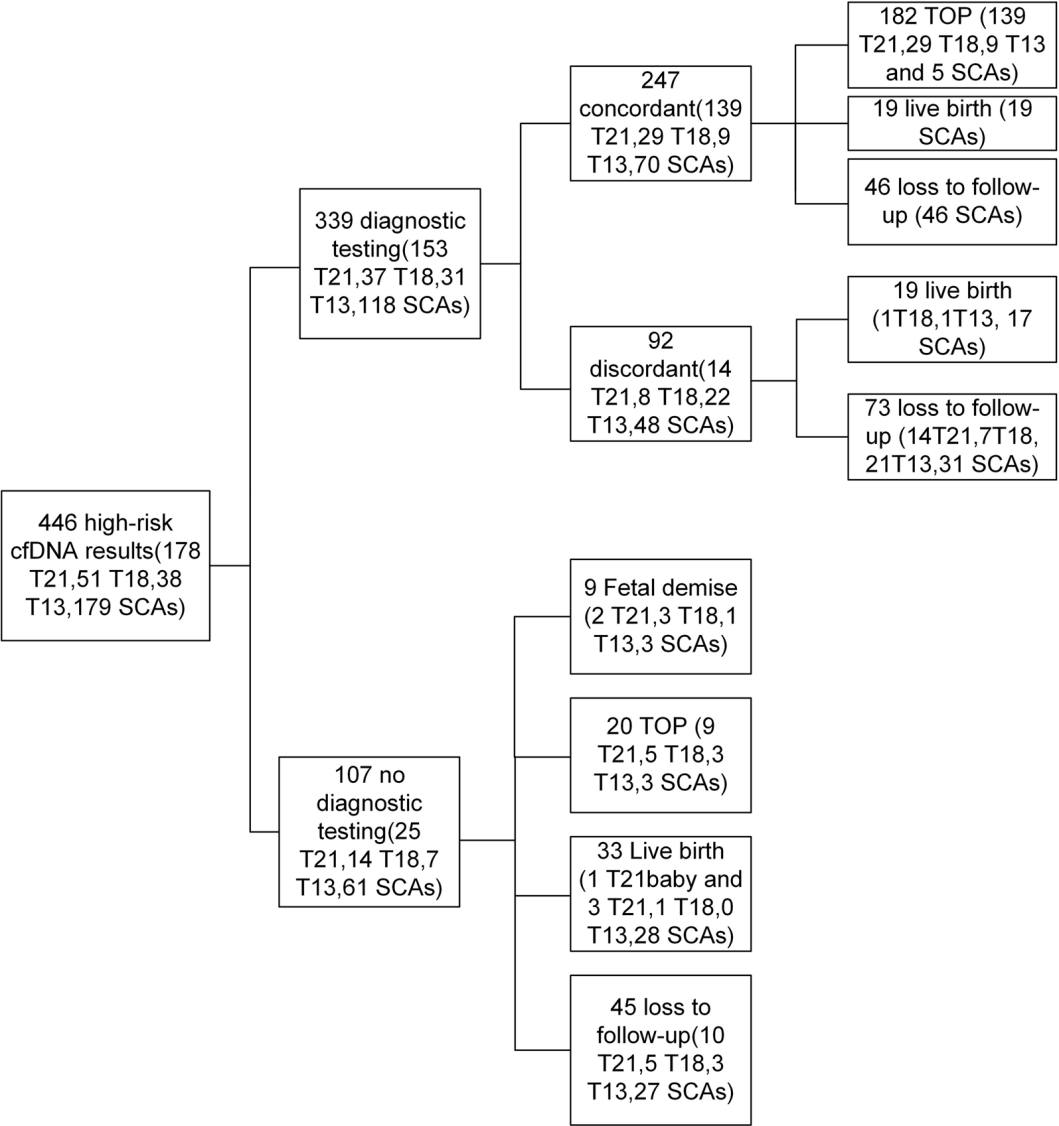
Detailed NIPT results, diagnostic test results, and outcome of pregnancy for the cases at high risk for common aneuploidies and SCAs. T21: Trisomy 21, T18: Trisomy 18, T13: Trisomy 13, SCAs: sex chromosomal aneuploidies, TOP: termination of pregnancy

The 446 high risk cases comprised 178 T21, 51 T18, 38 T13, and 179 SCAs (82 cases of 45,X; 25 cases of 47,XXX; 48 cases of 47,XXY; and 24 cases of 47,XYY). Three hundred and thirty nine pregnant women underwent amniocenteses, and 139 T21, 29 T18, 9 T13, and 70 SCAs (13 cases of 45,X; 9 cases of 47,XXX; 32 cases of 47,XXY; and 16 cases of 47,XYY) cases were confirmed by karyotype analysis (Fig. 1). A total of 73.68% (182/247) of the women chose to terminate their pregnancies. Nineteen women had live births, giving birth to 19 babies with SCAs (4 cases of 45,X; 10 cases of 47,XXY; and 5 cases of 47,XYY). Ninety two cases including 14 T21, 8 T18, 22 T13, and 48 SCAs (35 cases of 45,X; 3 cases of 47,XXX; 5 cases of 47,XXY; and 5 cases of 47,XYY) with confirmatory testing results showed nonconcordant karyotyping results. Of the 92 cases, 87 cases showed normal karyotype analysis results, while 5 cases had different chromosomal abnormalities (Additional file 1: Table 1). Nineteen cases had normal live births. The other 73 cases had no confirmed pregnancy outcomes (Fig. 1). Of 107 high-risk cases without confirmative invasive tests, 20 women chose termination of pregnancy (TOP) because of abnormal ultrasound findings or anxiety (Table 1, Fig. 1). Nine cases (2 cases of T21, 3 cases of T18, 1 case of T13, 2 case of XO, and 1 case of XXY) lost pregnancies due to fetal demise. Thirty-three pregnant women continued their pregnancies and had normal live births with normal neonatal examination results and physical appearance at the time of postnatal follow‐up (Fig. 1).

**Table 1:** Abnormal ultrasound findings in pregnancies without confirmed high-risk expanded NIPT results

CaseID	Age	Gestational week	Expanded NIPT result	Abnormal ultrasound result	Pregnancy outcome
1	37	18w + 2	T21	(1) Nasal bone is not shown; (2) possible complete endocardial pad defect; (3) middle finger phalanx is not shown	TOP
2	32	18w + 1	T21	Possible T21: (1) the middle phalanx of the left little finger is not shown, the phalanx in the right little finger is punctate calcification; (2) the ventricular septal defect (possible malformation); (3) the nasal bone dysplasia; (4) the flap angle is increased; (5) the right foot is changed similar to a "shoe foot"	TOP
3	28	19w + 4	T21	Possible T21: (1) nasal bone is not shown; (2) Increased flap angle; (3) thickening of liver parenchyma; (4) widening of umbilical vein ventral section; (5) abdominal circumference, femoral diameter and humeral diameter are smaller normal than gestational age; 6) abnormal intracardiac structure	TOP
4	36	17w + 4	T21	Possible T21: (1) nasal bone is not shown; (2) increased flap angle; (3) the little finger phalanx of the hands is not shown; (4) multiple echoes in the abdominal cavity; (5) abnormal intracardiac structure	TOP
5	24	21w + 4	T21	Possible T21: (1) "double bubble sign" in the abdominal cavity; (2) fetal nasal bone loss; (3) liver echo thickening; (4) bilateral femur and tibia less than -2 standard deviations lower than normal for gestational age; (5) increased flap angle; (6) abnormal intracardiac structure	TOP
6	34	19w + 3	T21	NA	TOP
7	36	18	T21	NA	TOP
8	39	12	T21	NA	TOP
9	43	13	T21	NA	TOP
10	30	18w + 6	T18	Possible T18: (1) the left cleft lip (degree III) and splitting; (2) ventricular septal defect; (3) possible right hand overlap; (4) "strawberry" head	TOP
11	39	17w + 5	T18	Possible T18: (1) no-leaf full forebrain malformation; (2) fetal central cleft lip (degree III) with cleft palate; (3) nasal bone is not seen; (4) narrow eye distance; (5) middle finger phalanx of both hands is not shown; (6) abnormal cardiac structure	TOP
12	35	14w + 6	T13	Possible T13: (1) forebrain noncracking malformation; (2) narrow eye distance; (3) strong echogenic plaque in bilateral eyeballs; (4) median cleft lip (class III), cleft palate; (5) slightly enhanced echogenicity of both kidney parenchyma; (6) abnormal intracardiac structure possible; (7) poor appetite filling; (8) multiple strong echogenic spots in the abdominal cavity	TOP
13	40	19w + 3	T13	(1) Narrow eye distance, single nostril; (2) fetal intracardiac structural abnormalities; (3) multifinger after both hands; (4) enhanced bowel echo	TOP
14			T18	NA	TOP
15			T18	NA	TOP
16	38	16	T18	NA	TOP
17	38	17w + 1	XXY	Fetal cleft lip and alveolar cleft	TOP
18	43	13w + 1	T13	Intrauterine single fetus, NT 1.07 MM	TOP
19	28	19w + 5	XO	NA	TOP
20	28	18w + 1	XO	(1) Fetal femur diameter smaller than normal for gestational age; (2) fetal umbilical cord is wrapped around the neck	

T21: Trisomy 21, T18: Trisomy 18, T13, Trisomy 13, NT: Nuchal translucency, NA: not available

### Expanded NIPT results for RATs and CNVs

**Fig. 2:**
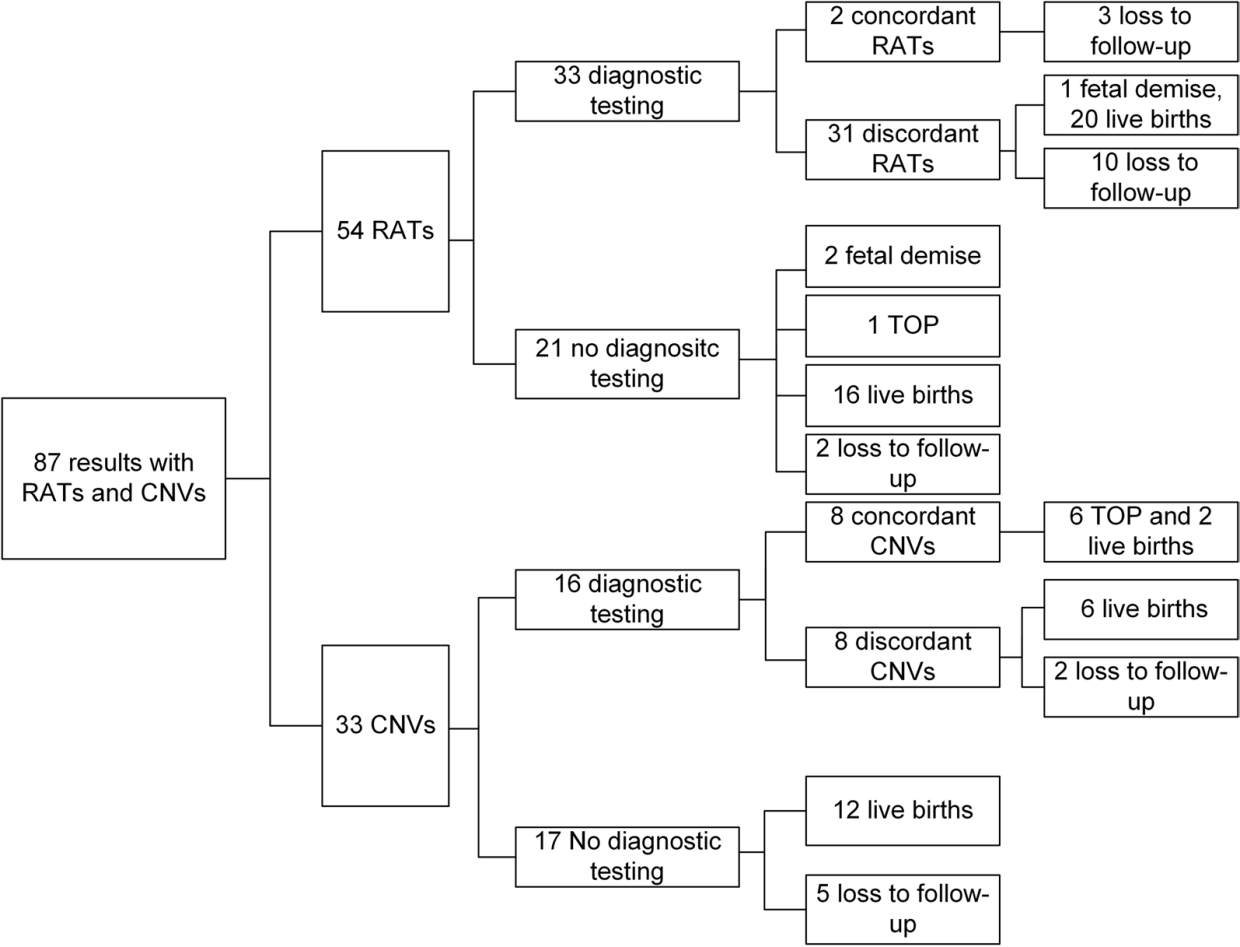
Detailed NIPT results, diagnostic test results, and outcomes of pregnancy for cases at high risk for RATs and CNVs. RATs: rare autosomal aneuploidies, CNVs: copy number variations

Fifty-four RAT cases and 33 CNV cases were reported among the 24,702 cases. Of the 54 RAT cases, Trisomy 8 (T8), Trisomy 14 (T14), and Trisomy 16 (T16) were the top three most frequent RATs, with frequencies of 16.7% (9/54), 13% (7/54), and 13% (7/54), respectively (Additional file 2: Figure. 1, Additional file 1: Table 2). Thirty-three women with RATs underwent amniocenteses. Two cases of T14, Trisomy 22 (T22) were confirmed by karyotype analysis, and the other 31 cases had normal karyotype results. The T22 case was mosaics, evidenced by karyotype results of 47,XX,+22[23]/46,XX[41]. Of the 31 discordant cases, 1 case (T9) lost the pregnancy due to fetal demise. Twenty pregnant women had normal live births with normal neonatal examination results and physical appearance at the time of postnatal follow‐up. The other 10 cases were lost to follow-up (Fig. 2). Twenty-one cases with RAT declined amniocenteses, 1 case directly selected TOP, and 2 cases (T14 and T16) lost pregnancies due to fetal demise. Sixteen pregnant women had normal live births with normal neonatal examination results and physical appearance at the time of postnatal follow‐up (Fig. 2).

Sixteen cases of the 33 CNVs underwent CMA tests, while the others declined confirmatory diagnostic tests. Eight CNVs of the 16 cases underwent confirmatory testing, and the results were concordant with CMA results (Additional file 1: Table 3); 6 of the CNV cases underwent TOP, and 2 cases had normal live births. Eight cases showed normal results in CMA analysis, and 6 cases had normal live births. In the CNV cases with diagnostic testing, two cases had two imbalanced translocations, with only one of the two detected in NIPT, suggesting a limitation of NIPT technology in detecting imbalanced translocations. Among the 17 cases without invasive diagnostic testing, 12 had normal live births and 5 cases were lost to follow-up (Fig. 2, Additional file 1: Table 4).

### Low‐risk expanded NIPT results

**Fig. 3:**
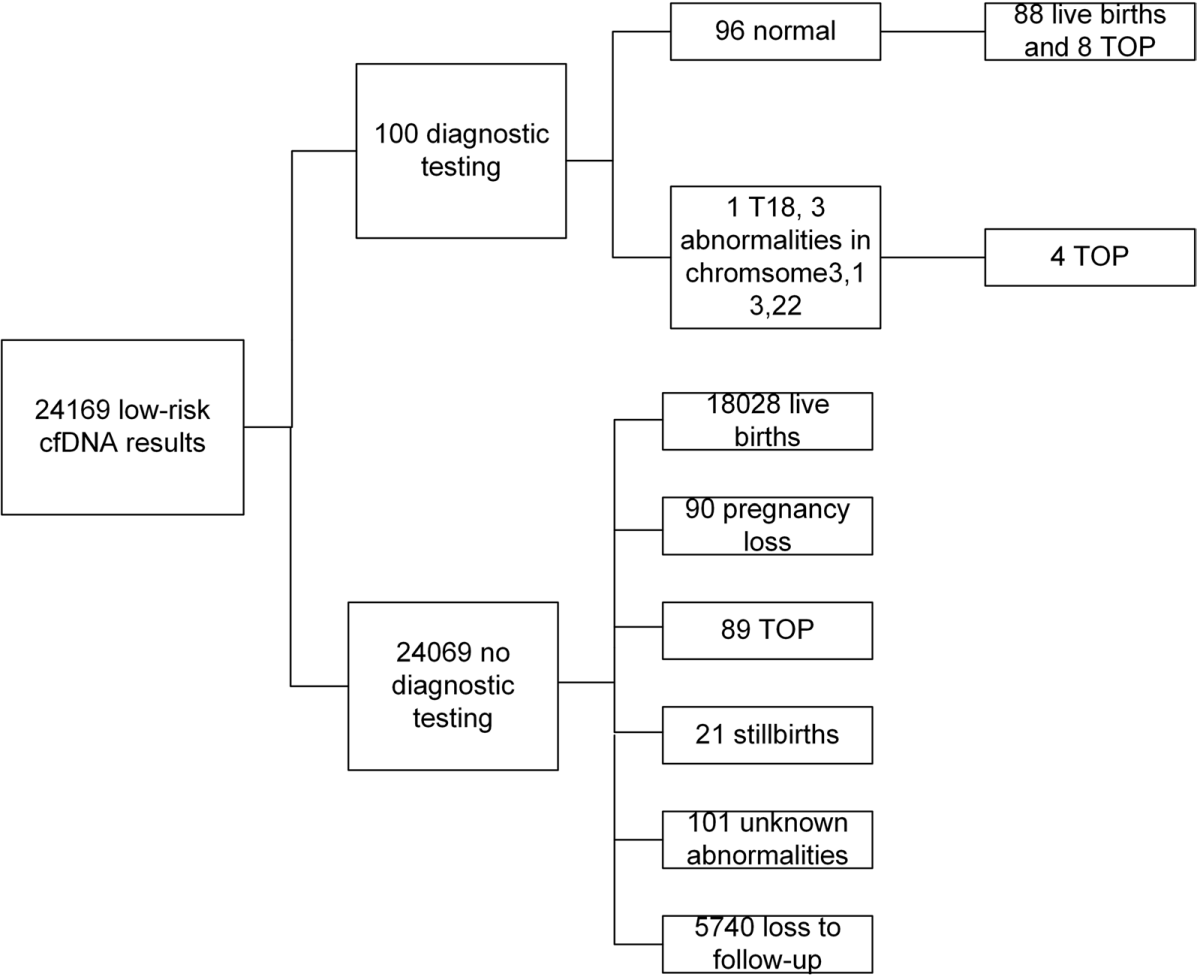
Detailed NIPT results, diagnostic test results, and outcomes of pregnancy for low-risk cases

Of the 24,169 low-risk cases, 76.25% (18,429/24,169) of the cases were successfully followed. One hundred cases underwent diagnostic tests due to anxiety and pressure. A total of 24,069 cases did not have amniocenteses, and 5740 cases were lost to contact. Among the 100 cases with diagnostic test results, 88 cases showed normal karyotype results and gave normal births and 8 women chose TOP because of fetal anatomical anomalies detected by ultrasound. One false negative case of T18 and three abnormalities in chromosome 3 [(CMA, arr[hg19]3p26.3p25.3[61,891–10,914,685] × 1), 13 (Karyotype analysis, 46,XY,r(13)(p12q34)[69]/45,XY,-13[7]/46,XY,dic r(13;13)(p12q34;p12q34)[2]), 22 (CMA, arr[hg19]22q13.2q13.33[44,088,529-51,197,766] × 1)] were diagnosed, and the four cases terminated their pregnancies (Fig. 3). Of the 24,069 cases who had low-risk results and did not receive diagnostic testing, 18,028 had normal live births, 90 lost the pregnancy, 89 underwent TOP because of aberrant fetal ultrasound results, 21 had stillbirths, and 101 reported unknown fetal abnormalities (Fig. 3).

### Performance of expanded NIPT

On the basis of expanded NIPT results and the available pregnancy outcome data, we analyzed the performance of expanded NIPT in detection of common aneuploidies, SCAs, RATs and CNVs (Table 2). The sensitivity was 100% for T21, 96.67% for T18, and 100% for T13; the specificity was 99.92% for T21, 99.96% for T18, and 99.88% for T13; the positive predictive value (PPV) was 90.85% for T21, 78.38% for T18 and 29.03% for T13; and the negative predictive value (NPV) was over 99.99% for common aneuplodies. Expanded NIPT detected SCAs, RATs and CNVs with PPVs of 59.32%, 6.45%, and 50%, respectively based on available data. Notably, the PPV for individual SCAs was as follows: 45,X 25.49% (13/51), 47,XXX 75% (9/12), 47,XXY 94.12% (32/34), and 47,XYY 76.19% (16/21) (Table 2). When stratified by CNV size, expanded NIPT detected eight cases with CNV size was greater than 10 Mb and eight samples with CNV size was less than 10 Mb. The PPV for individual CNVs > 10 Mb was 62.5%, but the performance decreased to 42.86% when the size of CNVs was smaller than 10 Mb (Table 2). We chose all the samples with first trimester and found 949 cases in our study. The PPVs for common aneuploidies, SCAs, RATs and CNVs were calculated and presented in the Additional file 1: Table 5. The results of first trimester screening were not comparable to NIPT, due to limited number of samples at high risk for different NIPT results. The possible false positive cases in our study comprised 92 common trisomies and SCAs, 30 rare trisomies, 8 CNVs, 4 cases which showed low-risk NIPT results but diagnosed with T18 or CNVs and unknown 301 cases who may have had trisomies in fetus but not in placenta due to placental mosaics; Thus, the overall false positive rate of expanded NIPT is at least 2.35% (435/18,516).

**Table 2:** The performance of expanded NIPT in screening for T21, T18, T13, SCAs, RATs, and CNVs

Expanded NIPT result	TP	FP	TN	FN	Sensitivity (95% CI)	Specificity (95% CI)	PPV (95% CI)	NPV (95% CI)
T21	139	14	18,363	0	100% (97.38–100%)	99.92% (99.87–99.96%)	90.85% (85.47–94.37%)	100.00%
T18	29	8	18,478	1	96.67% (82.78–99.92%)	99.96% (99.91–99.98%)	78.38% (64.38–87.91%)	99.99% (99.96–100%)
T13	9	22	18,485	0	100% (66.37–100.00%)	99.88% (99.82–99.93%)	29.03% (21.22–38.32%)	100.00%
T21 (phenotype)	146	17					91.25%	
T18 (phenotype)	34	9					79.07%	
T13 (phenotype)	12	22					35.29%	
SCAs	70	48					59.32%	
SCAs (phenotype)	72	62					53.73%	
XO	13	38	–	–			25.49%	
XO (phenotype)	15	52					22.39%	
XXX	9	3					75%	
XXY	32	2					94.12%	
XYY	16	5					76.19%	
RATs	2	31	–	–			6.45%	
CNVs	8	8	–	–			50%	
CNVs (< 10 M)	3	4					42.86%	
CNVs (> 10 M)	5	4					62.5%	

TP: true positive confirmed by genetic test, FP: false positive confirmed by genetic test, TN: true negative confirmed by clinical follow-up, FN: false negative confirmed by clinical follow-up. PPV: positive predictive value, NPV: negative predictive value. PPVs for T21, T18, T13, SCAs, and XO were confirmed based on cytogenetic test. PPVs for T21 (phenotype), T18 (phenotype), T13 (phenotype), SCAs (phenotype), and XO (phenotype) were calculated based on prenatal ultrasound detection and cytogenetic tests. Fetal demise and fetal anomalies revealed by prenatal ultrasound were considered possible T21, T18, T13, XO. T21, and T18 were excluded based on normal newborn examination

### Comparison of prenatal diagnosis willingness and pregnancy outcomes

**Fig. 4:**
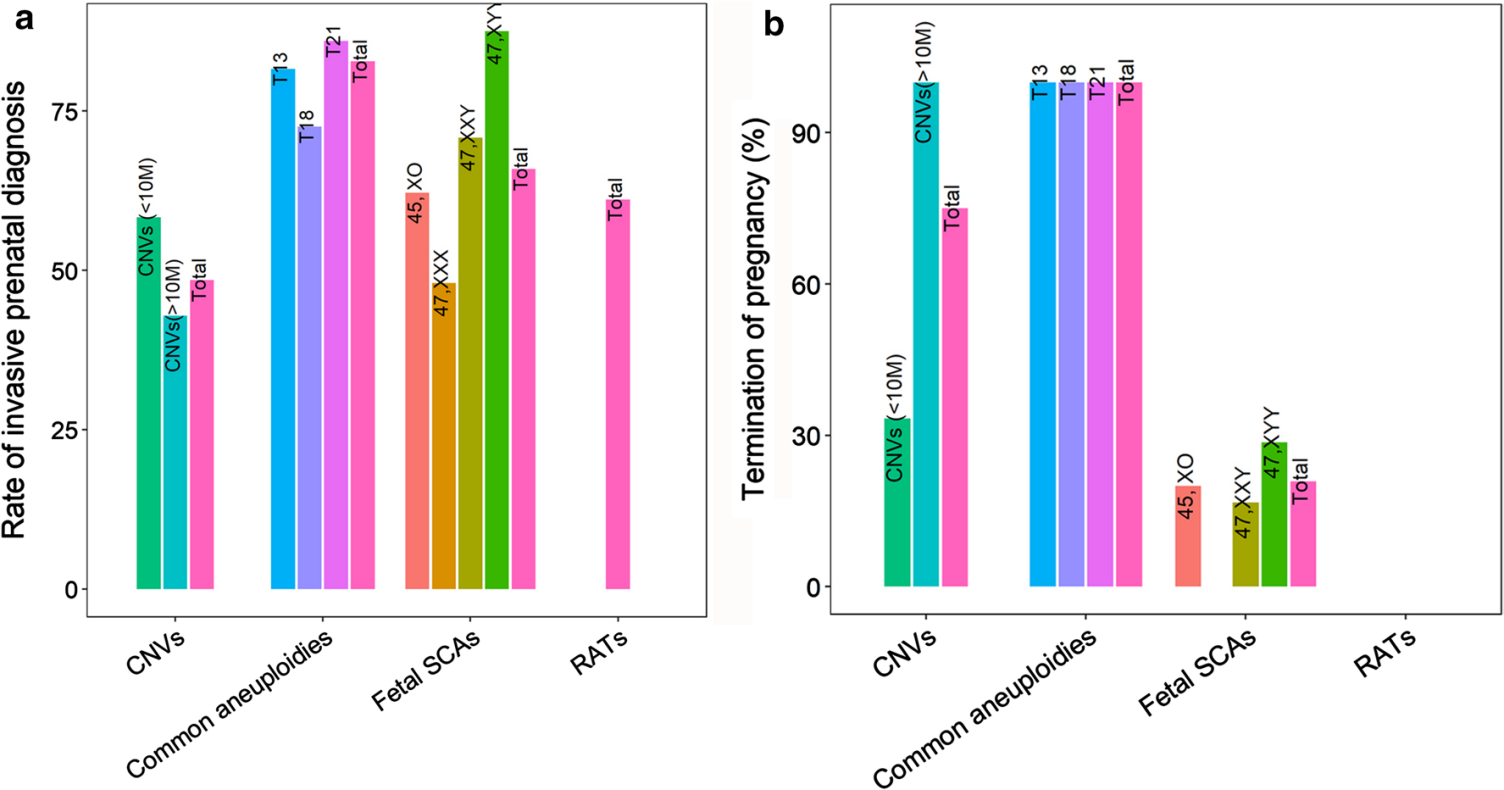
Comparison of prenatal diagnosis willingness and pregnancy outcomes. **a** The rate of prenatal diagnosis for different women with high-risk expanded NIPT results. **b** The rate of termination of pregnancy for different women with high-risk expanded NIPT results

Of 267 women at high risk for common aneuploidies, 221 women (82.77%) accepted the invasive test, and 46 women (17.23%) declined the invasive test for various reasons, such as severe ultrasound abnormalities, concerns about abortion, etc. As shown in Table 3 and Fig. 4, the rate of prenatal diagnosis for women with high-risk results for common fetal aneuploidies was significantly higher than those with high-risk results for SCAs (65.92%, 118/179), RATs (61.11%, 33/54), and CNVs (48.48%, 16/33) (Fisher’s exact test, *p* < 0.05 for all cases). No significant difference in the rate of invasive prenatal diagnosis was observed among the women at high risk for SCAs, RATs, and CNVs (Fisher’s exact test, *p* > 0.05 for all cases). A total of 100% (181/181) of the women carrying fetuses with T21/T18/T13 terminated their pregnancy, which was significantly higher than those at high risk for SCAs (20.83%, 5/24) and CNVs (75%, 6/8) (Fisher’s exact test, *p* < 0.05 for all cases). Women carrying fetuses with 47,XXY and CNVs (< 10 M) were more likely to continue their pregnancy (Table 3, Fig. 4).

**Table 3:** Comparison of prenatal diagnosis willingness and pregnancy outcomes

Group	Number of high-risk cases	Accepted invasive testing	Declined invasive testing	Number of confirmed cases	Terminate pregnancy	Continue pregnancy
Common aneuploidies	267	221(82.77%)	46(17.23%)	177	177(100%)	0(0%)
T21	178	153(85.96%)	25(14.04%)	139	139(100%)	0(0%)
T18	51	37(72.55%)	14(27.45%)	29	29(100%)	0(0%)
T13	38	31(81.58%)	7(18.42%)	9	9(100%)	0(0%)
Fetal SCAs	179***	118(65.92%)	61(34.08%)	24***	5(20.83%)	19(79.17%)
45,X	82***	51(62.2%)	31(37.8%)	5***	1(20%)	4(80%)
47,XXX	25***	12(48%)	13(52%)	13	NA	NA
47,XXY	48	34(70.83%)	14(29.17%)	12***	2(16.67%)	10(83.33%)
47,XYY	24	21(87.5%)	3(12.5%)	7***	2(28.57%)	5(71.43%)
RATs	54***	33(61.11%)	21(38.89%)	3	NA	NA
CNVs	33***	16(48.48%)	17(51.52%)	8***	6(75%)	2(25%)
CNVs (< 10 M)	12*	7(58.33%)	5(41.67%)	3***	1(33.33%)	2(66.67%)
CNVs(> 10 M)	21***	9(42.86%)	12(57.14%)	5	5(100%)	0

*< 0.05, ***< 0.005, Fisher’s exact test. The differences in the rate of prenatal diagnosis and termination of pregnancy were compared between the common aneuploidies group and any other group

## Discussion

In this study, 24,702 pregnant women were enrolled to investigate the clinical performance of expanded NIPT. The sensitivity and specificity of expanded NIPT was over 99% for T21, T18, and T13, which was consistent with previous studies [7–11]. Our results presented a comparable PPV for T21 and T18 but a relatively low PPV for T13 compared to other studies [7, 15]. This could be because of the small number of cases with high risk T13 results in our study; the low number of T13 cases would increase the variation of PPV. Only 2.16% (533/24,702) of cases were classified as high risk for common aneuploidies, SCAs, RATs, CNVs and needed to undergo diagnostic karyotype analysis. The application of expanded NIPT prevented nearly 98% of invasive diagnostic procedures and reduced unnecessary fetal losses caused by invasive tests. The miscarriage rates were 0.8–1.5% following invasive fetal karyotyping procedures [16, 17]. In 18.69% (20/107) of cases with unconfirmed high-risk expanded NIPT results, fetal anatomical anomalies were reported by regular ultrasound examination, and these women elected for TOP based on the abnormal ultrasound results. Ultrasound examination revealed 4 suspicious T21, 2 suspicious T18, and 1 suspicious T13 in the 20 cases. These results demonstrated that the number of true-positive cases might have been underestimated in this study.

In our study, expanded NIPT detected SCAs with a PPV of 59.32%, which is similar to that reported by Porreco’s study [18] but much lower than the results reported by other studies [4, 5]. The difference is largely attributable to the different analytical techniques used in the studies. Unlike the z-scores algorithm used in our study and Porreco’s study [18], Mazloom constructed a classification algorithm for SCA detection and determined the accuracy of the classification algorithm on another independent cohort of maternal samples [4]. Hooks et al. developed a chromosome-selective approach and devised a risk algorithm incorporating fetal fraction to evaluate the risk for SCAs [5]. In our study, expanded NIPT more accurately detected triple X, XXY, and XYY syndrome than XO syndrome, which is in line with findings reported by others [15, 19, 20]. The difference in the detection of different SCAs can be ascribed to two main causes. First, there are 58 homologous genes on the X and Y chromosome, and 29 genes are located at the ends of sex chromosomes. The sequencing length of expanded NIPT is as short as 36 bases; therefore, errors in sequencing these homologous genes might easily occur on the X and Y chromosomes [19]. Second, X chromosome mosaicism is another contributing maternal factor to the difference in pregnant women [21].

In recent years, expanded NIPT has been applied to screening for other chromosomal abnormalities including fetal RATs and CNVs [22, 23]; however, it remains unclear regarding the detection accuracies of RATs and CNVs of the expanded NIPT, the application of expanded screening is still under debate in clinical settings. In our clinical practice, the PPV was 6.45% for the detection of RATs and 50% for the detection of CNVs. The PPV of expanded NIPT for RATs was as low as 6.45%, which is similar to Karuna’s study [24]. The majority (94%) of the RATs identified by NIPT were discordant with confirmative results, most likely due to confined placental mosaicism. Confined placental mosaicism is associated with a wide range of adverse fetal and maternal outcomes, such as multiple congenital anomalies, preterm birth, fetal growth restriction and stillbirth [25–27]. Regular ultrasound monitoring of fetal growth is recommended for all the cases at high risk for RATs.

By comparing the set of CNVs with the Online Mendelian Inheritance in Man database, 4 CNVs cases were pathogenic and 3 CNVs were unknown (Additional file 1: Table S3). The results suggested the screening test was capable of detecting fetal RATs and CNVs, but it resulted in a large fraction of false-positive cases. Previous studies have claimed benefits [28]; however, we suggest that expanded NIPT has not demonstrated a sufficient true-positive rate in the detection of RATs and CNVs, particularly for RATs. Therefore, the application of expanded NIPT to screening for RATs and CNVs must be balanced with the miscarriage risk of using diagnostic testing due to the procedure itself.

There are many factors which affect the willingness of pregnant women to undertake invasive prenatal diagnosis and TOP upon receiving different high-risk expanded NIPT results. Furthermore, the attitude towards different high-risk expanded NIPT results remains unknown. In this study, in comparison to women with high-risk results for SCA, RAT and CNV, the rate of prenatal diagnosis for women with high-risk T21/T18/T13 results was significantly increased. These findings were in line with those of Zhou’s study [12]. The pregnant women at high risk for T21/T18/T13 are more likely to take prenatal diagnosis tests because they know more about these three diseases. Due to the seriousness of the diseases, all pregnant women are willing to undergo prenatal diagnosis, and almost all of the high-risk cases in China will terminate their pregnancy [12]. Zhou’s study reported that women at high risk for Turner syndrome were more willing to continue their pregnancies, while we found women carrying fetuses with 47,XXY tended to continue their pregnancies while women with other fetal SCAs were more likely to underdo TOP. Women with confirmed larger CNVs (> 10 M) showed higher TOP rates than those with confirmed smaller CNVs (< 10 M). These differences may be related to prenatal genetic counseling, lower perceived clinical significance, and the cognition level of pregnant women. The restrictions and regulations for TOP in China are strict, especially in late pregnancy. To terminate a pregnancy over 14 weeks of gestation, it is generally necessary to go to a designated prenatal diagnostic institution and discuss it with doctors. Multidisciplinary consultation is usually required for TOP over 28 weeks of gestation. The results of our study might provide guidance for clinicians as to how to address pregnant women with different types of high-risk expanded NIPT tests in late pregnancy. For instance, women confirmed to be high-risk for common aneuploidies and SCAs are highly recommended to undergo TOP. While, the treatment for women with high-risk CNV results (< 10 M) might be cautious, the severeness of the disease and parental willingness are needed to be taken into account.

Despite the significant findings obtained, this study has certain limitations. First, participants who did not have confirmatory diagnostic results, low-risk cases with pregnancy losses, cases with TOP, stillbirths, cases with unknown abnormalities, and cases lost to follow-up were excluded from the calculation of the NIPT accuracy in the study, which might impact the accuracy of results and ultimately the conclusions of our study. Second, the true-negative cases were low risk in expanded NIPT and validated by normal neonatal physical examination except for SCAs or diagnostic test analysis. The lack of an insurance claim is not robust enough evidence that a false-positive result did not occur, and this might affect the performance evaluation of expanded NIPT. Third, the findings and conclusions were obtained from a retrospective study, we acknowledge the risk of ascertainment bias associated with retrospective identification of cases. Last, the findings and conclusions are based on a cohort of 24,702 cases from a single tertiary center and may have regional bias. Therefore, further work might be needed to validate the results using data from multiple independent centers.

## Conclusion

In conclusion, our study demonstrates that expanded NIPT detects fetal trisomies 21,18, and 13 with high sensitivity and specificity but shows a relatively low true-positive value for SCAs, RATs, and CNVs. The application of expanded NIPT to detect SCAs, RATs, and CNVs, particularly RATs, should be considered with caution. After receiving a high-risk expanded NIPT result, women at high risk for common trisomies are more likely to pursue invasive prenatal diagnosis and terminate their pregnancies. The results of this study may provide solid evidences that guide genetic counselling and decision making in the treatment of different types of high-risk expanded NIPT results in clinical settings.

## Supplementary Information

**Additional file 1.** **Table S1.** The 92 cases with discordant results between NIPT test and karyotype analysis results; **Table S2.** The summary of NIPT results, karyotype analysis results and pregnancy outcomes of 54 RAT cases; **Table S3.** The NIPT, karyotype and CMA results of 16 cases with CNVs; **Table S4.** The NIPT, karyotype and CMA results of 17 cases at high risk for CNVs without diagnostic results.

**Additional file 2**: Supplementary Figure 1. Frequencies of RATs in 54 cases with high risk RAT results and 3 true-positive RAT cases. M15: monosomy 15, M4: monosomy 4, T17: Trisomy 17, T4: Trisomy 4, T5: Trisomy 5, M14: monosomy 14, T10: Trisomy 10, T11: Trisomy 11, T15: Trisomy 15, T20: Trisomy 20, T3: Trisomy 3, T9: Trisomy 9, T2: Trisomy 2, T6: Trisomy 6, T22: Trisomy 22, T16: Trisomy 16, T14: Trisomy 14, T8: Trisomy 8.

## Data Availability

All data generated or analyzed during this study are included in this published article and its supplementary files.

## Abbreviations

Expanded NIPT: Expanded noninvasive prenatal test
T21: Trisomy 21
T18: Trisomy 18
T13: Trisomy 13
SCAs: Sex chromosomal aneuploidies
RATs: Rare autosomal aneuploidies
CNVs: Copy number variations
TOP: Termination of pregnancy
CMA: Chromosomal microarray analysis
TP: True positive
TN: True negative
PPV: Positive predictive value
NPV: Negative predictive value
NT: Nuchal translucency
