# Supplementary material for: Expanded noninvasive prenatal testing for fetal aneuploidy and copy number variations and parental willingness for invasive diagnosis in a cohort of 18,516 cases
Source: BMC Med Genomics. 2021 Apr 14;14:106. doi: 10.1186/s12920-021-00955-6 (PMC8045328; doi:10.1186/s12920-021-00955-6)
Supplement: Supplementary file 1 — Additional file 1. Table S1. The 92 cases with discordant results between NIPT test and karyotype analysis results; Table S2. The summary of NIPT results, karyotype analysis results and pregnancy outcomes of 54 RAT cases; Table S3. The NIPT, karyotype and CMA results of 16 cases with CNVs; Table S4. The NIPT, karyotype and CMA results of 17 cases at high risk for CNVs without diagnostic results. [file 12920_2021_955_MOESM1_ESM.docx]

**Table S1. The 92 cases with discordant results between NIPT test and karyotype analysis results**

| NIPT\ Karyotype analysis | 46,XX/46,XY | 47,XXX | 47,XXY |
| --- | --- | --- | --- |
| T21 | 14 | 0 | 0 |
| T18 | 8 | 0 | 0 |
| T13 | 22 | 0 | 0 |
| SCA | 43 | 3 | 2 |

**Table S2. The summary of NIPT results, karyotype analysis results and pregnancy outcomes of 54 RAT cases**

| NIPT | Number of RATs in NIPT test | Number of cases with diagnostic results | Number of confirmed cases | 46,XX/46,XY | 47,XX,+6[1]/  46,XX[102] | 46,XX,der(14)  t(14;14)(p10;q31.1)dn | 47,XX,+22[23]/  46,XX[41] | Number of IUFD | Number of live births |
| --- | --- | --- | --- | --- | --- | --- | --- | --- | --- |
| T2 | 3 | 1 | 0 | 1 | 0 | 0 | 0 | 0 | 3 |
| T3 | 2 | 1 | 0 | 1 | 0 | 0 | 0 | 0 | 1 |
| T4 | 1 | 1 | 0 | 1 | 0 | 0 | 0 | 0 | 1 |
| M4 | 1 | 0 | 0 | 0 | 0 | 0 | 0 | 0 | 1 |
| T5 | 1 | 1 | 0 | 1 | 0 | 0 | 0 | 0 | 0 |
| M5 | 1 | 1 | 0 | 1 | 0 | 0 | 0 | 0 | 1 |
| T6 | 2 | 1 | 1 | 0 | 1 | 0 | 0 | 0 | 1 |
| T8 | 9 | 6 | 0 | 6 | 0 | 0 | 0 | 0 | 4 |
| T9 | 5 | 4 | 0 | 4 | 0 | 0 | 0 | 1 | 4 |
| T10 | 2 | 2 | 0 | 2 | 0 | 0 | 0 | 0 | 2 |
| T11 | 2 | 1 | 0 | 1 | 0 | 0 | 0 | 0 | 1 |
| T14 | 7 | 4 | 1 | 3 | 0 | 1 | 0 | 1 | 2 |
| M14 | 2 | 1 | 0 | 1 | 0 | 0 | 0 | 0 | 2 |
| T15 | 2 | 0 | 0 | 0 | 0 | 0 | 0 | 0 | 2 |
| T16 | 6 | 3 | 0 | 3 | 0 | 0 | 0 | 1 | 4 |
| M16 | 1 | 1 | 0 | 1 | 0 | 0 | 0 | 0 | 1 |
| T17 | 1 | 1 | 0 | 1 | 0 | 0 | 0 | 0 | 1 |
| T20 | 1 | 0 | 0 | 0 | 0 | 0 | 0 | 0 | 1 |
| T22 | 5 | 4 | 1 | 3 | 0 | 0 | 1 | 0 | 3 |
| Total | 54 | 30 | 3 | 27 | 1 | 1 | 1 | 3 | 35 |

IUFD denotes intra-uterine fetal death.

**Table S3. The NIPT, karyotype and CMA results of 16 cases with CNVs**

| **Case ID** | **CNV type** | **NIPT test result** | **Karyotype result** | **CMA result** | **Pathogenicity** |
| --- | --- | --- | --- | --- | --- |
| 1 | Duplication | dup(4)(q12-q13.1);size:5.48M | 46,XY | Normal |  |
| 2 | Deletion | del(3)(q13.32-q25.31);size:37.63M | 46,XY,inv(9)(p11q13) | Normal |  |
| 3 | Deletion | del(2)(p25.1-p24.1);size:13.06M | 46,XX | Normal |  |
| 4 | Duplication | dup(12)(p13.33-p11.1);size:33.69M | mos 47,XY,+12[4]/46,XN[67] | arr [hg19]12p11.1q12(34,257,325-38,881,750)x3 | Pallister-Killian 综合征 |
| 5 | Duplication | dup(7)(q21.13-q36.3);size:68.90M | 46,XY,del(7)(q32) | arr [hg19]7q32.3q36.3(131,443,470-159,119,707)x1 |  |
| 6 | Duplication | dup(4)(q12-q13.1);size:5.51M | 46,XY | arr [hg19]4q12q13.1(58,193,591-62,730,657)x3 |  |
| 7 | Deletion | del(13)(q33.2-q34); size:8.53M | 46,XX,del(13)(q-33.1)dn | arr [hg19]13q33.1q34(104,569,091-115,107,733)x1 |  |
| 8 | Deletion | del(16)(p13.3-p13.2); size:5.03M | 46,XX | arr [hg19]4q35.2(189,770,374-190,957,460)x1 | Pathogenicity unknown |
| 9 | Deletion | del(18)(p11.32-p11.22);size:8.75M | 46,XY,del(18)(p11.22) | arr [hg19]16q24.1q24.3(84,925,809-90,155,062)x3,18p11.32p11.22(136,227-9,714,948)x1 | Pathogenicity unknown |
| 10 | Duplication | dup(11)(q23.3-q25);size:17.66M | 47,XX,+der(22)t(11;22)(q23;q11.2) | arr [hg19]11q23.3q25(116,683,754-134,937,416)x3,22q11.1q11.21(16,888,899-20,312,661)x3 | Emanuel-syndrome |
| 11 | Duplication | dup(3)(q22.1-q29);size:64.90M | 46,XX,der(9)t(3;9)(q23;p24) | arr [hg19]3q22.1q29(133,287,159-197,851,444)x3,9p24.3p24.2(208,454-3,889,747)x1 | , 3q29 microduplication syndrome |
| 12 | Deletion | del(11)(q21-q22.1);size:5.80M | 46,XY | Normal |  |
| 13 | Deletion | del(2)(q12.3-q13);size:5.89M | 46,XX | Normal |  |
| 14 | Deletion | del(6)(q15-q21);size:21.14M | 46,XX | Normal |  |
| 15 | Duplication | dup(11)(q14.3-q24.1);size:32.04M),del(11)(q24.2-q25);size:9.29M) | NA | arr [hg19]11q24.2q25(125,193,861-134,937,416)*1 | Jacobsen syndrome |
| 16 | Duplication | dup(11)(q11-q23.1);size:54.79M | 46,XY | Normal |  |

Note: del: deletion, dup: duplication, inv: inversion, t: translocation, der: derivative, NA: not available.

**Table S4. The NIPT, karyotype and CMA results of 17 cases at high risk for CNVs without diagnostic results**

| **Case ID** | **CNV type** | **NIPT test result** | **Karyotype result** | **CMA result** | **Pregnancy outcome** |
| --- | --- | --- | --- | --- | --- |
| 1 | Deletion | del(11)(p13-p12);size:5.41M | NA | NA | Normal live birth |
| 2 | duplication | dup(12)(q14.3-q24.33);size:63.86M;del(14)(q24.1-q32.33);size:38.64M | NA | NA | NA |
| 3 | Deletion | del(1)(p33-p36.33);size:48.51M,dup(1)(p33-q41);size:169.14M,del(1)(q41-q43);size21.73M | NA | NA | Normal live birth |
| 4 | Duplication | dup(4)(q12-q13.1);size:5.50M | NA | NA | Normal live birth |
| 5 | Deletion | del(14)(q21.3-q31.3);size:36.78M | 46,XY | NA | NA |
| 6 | Duplication | dup(11)(q14.3-q25);size:42.11M | NA | NA | Normal live birth |
| 7 | Duplication | dup(15)(q13.2-q14);size:5.01M | 46,XY | NA | Normal live birth |
| 8 | Deletion | del(7)(q1.12-q2.3);size:5.80M | NA | NA | NA |
| 9 | Duplication | dup(1)(q12-q32.3);size:70.76M;,del(1)(q41-q43);size:23.33M | 46,XX | NA | Normal live birth |
| 10 | Deletion | del(5)(p15.33-p15.2);size:10.36M | NA | NA | Normal live birth |
| 11 | Duplication | dup(12)(p13.33-p11.1);size:33.69M | NA | NA | Normal live birth |
| 12 | Deletion | del(20)(q12-q13.32);size:17.78M | NA | NA | Normal live birth |
| 13 | Deletion | del(20)(q11.21-q13.2);size:20.93M | NA | NA | NA |
| 14 | Deletion | del(20)( q11.21-q13.33);size:32.5M | 46,XX | NA | NA |
| 15 | Duplication | dup(9)(q12-q21.11)size:6.43M | NA | NA | Normal fetal ultrasound results and normal live birth |
| 16 | Duplication | dup(13)(q22.1-q34);size:40.56M;del(14)(q21.2-q32.33);size:61.51M | NA | NA | Normal live birth |
| 17 | Deletion | del(20);size:20M | 46,XY | NA | Normal live birth |

**Table S5.The performance of expanded NIPT in the detection of different types of NIPT results in the samples with first trimester**

| NIPT results | PPV |
| --- | --- |
| T21 | 100%(5/5) |
| T18 | 0 |
| T13 | 100%(1/1) |
| SCAs | 60%(3/5) |
| RATs | 100%(1/1) |
| CNVs | 100%(1/1) |
