# Supplementary figures and images for: Expanded noninvasive prenatal testing for fetal aneuploidy and copy number variations and parental willingness for invasive diagnosis in a cohort of 18,516 cases
Source: BMC Med Genomics. 2021 Apr 14;14:106. doi: 10.1186/s12920-021-00955-6 (PMC8045328; doi:10.1186/s12920-021-00955-6)

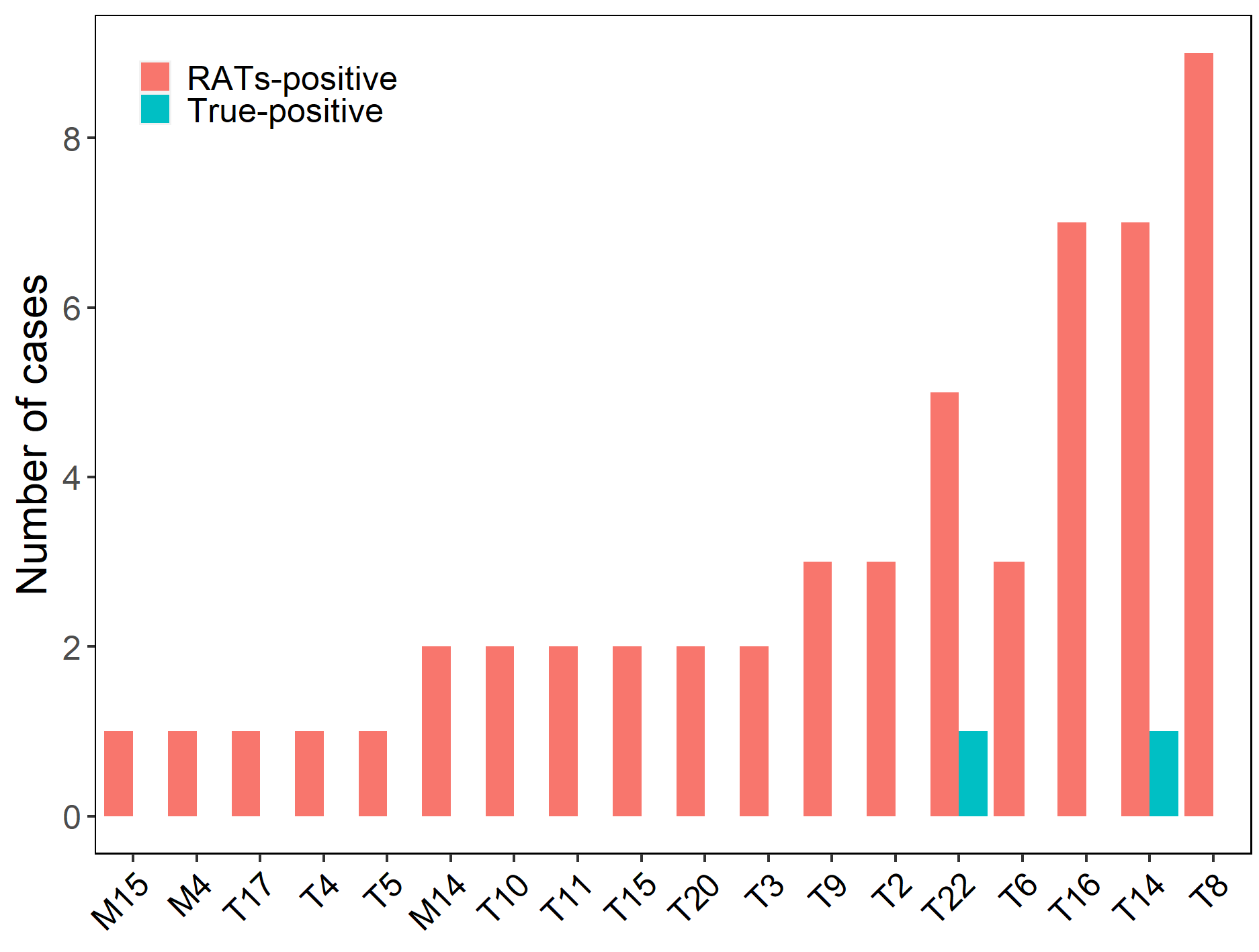

Supplement: Supplementary file 2 — Additional file 2: Supplementary Figure 1. Frequencies of RATs in 54 cases with high risk RAT results and 3 true-positive RAT cases. M15: monosomy 15, M4: monosomy 4, T17: Trisomy 17, T4: Trisomy 4, T5: Trisomy 5, M14: monosomy 14, T10: Trisomy 10, T11: Trisomy 11, T15: Trisomy 15, T20: Trisomy 20, T3: Trisomy 3, T9: Trisomy 9, T2: Trisomy 2, T6: Trisomy 6, T22: Trisomy 22, T16: Trisomy 16, T14: Trisomy 14, T8: Trisomy 8. [file 12920_2021_955_MOESM2_ESM.tiff]
